# The prevalence of adenoviral conjunctivitis at the Clinical Hospital of the State University of Campinas, Brazil

**DOI:** 10.6061/clinics/2015(11)06

**Published:** 2015-11

**Authors:** Roberto Damian Pacheco Pinto, Rodrigo Pessoa Cavalcanti Lira, Carlos Eduardo Leite Arieta, Rosane Silvestre de Castro, Sandra Helena Alves Bonon

**Affiliations:** I Universidade Estadual de Campinas (UNICAMP), Oftalmologia, Campinas/SP, Brazil; II Faculdade de Ciências Médicas e Laboratório de Virus, Campinas/SP, Brazil

**Keywords:** Adenovirus, Viral Conjunctivitis, Prevalence

## Abstract

**OBJECTIVES:**

Viral conjunctivitis is a common, highly contagious disease that is often caused by an adenovirus. The aim of this study was to evaluate the prevalence of adenoviral conjunctivitis by analyzing data from a prospective clinical study of 122 consecutively enrolled patients who were treated at the Clinical Hospital of the State University of Campinas (UNICAMP) after a clinical diagnosis of infectious conjunctivitis between November 2011 and June 2012.

**METHODS:**

Polymerase chain reaction was used to evaluate all cases of clinically diagnosed infectious conjunctivitis and based on the laboratory findings, the prevalence of adenoviral infections was determined. The incidence of subepithelial corneal infiltrates was also investigated.

**RESULTS:**

Of the 122 patients with acute infectious conjunctivitis included, 72 had positive polymerase chain reaction results for adenoviruses and 17 patients developed subepithelial corneal infiltrates (13.93%).

**CONCLUSIONS:**

The polymerase chain reaction revealed that the prevalence of adenoviral conjunctivitis was 59% in all patients who presented with a clinical diagnosis of infectious conjunctivitis from November 2011 to June 2012. The prevalence of adenoviral conjunctivitis in the study population was similar to its prevalence in other regions of the world.

## INTRODUCTION

Adenoviruses (AdV) are a major cause of viral conjunctivitis. They are responsible for 15% to 70% of all cases of conjunctivitis worldwide. These viruses belong to the *Mastadenovirus* genus of the *Adenoviridae* family, which is divided into six species (A - F) and 51 serotypes 1,2. Approximately one-third of human adenovirus serotypes are associated with common forms of adenoviral-related eye infections 3. The serotypes AdV8, 19 and 37 are often associated with epidemic keratoconjunctivitis (EKC), although other serotypes, such as AdV2, 3, 4, 5, 7, 10, 11, 21, 22, 29 and 34, have also been associated with this illness 4,5.

In general, viral conjunctivitis is diagnosed based on clinical features alone. The clinical presentation of the presumed viral conjunctivitis is characterized by acute symptoms, such as eye irritation, excessive tearing, soreness, foreign body sensations, light sensitivity and even blurred vision in advanced cases. Ocular findings include blepharedema, epiphora, conjunctival hyperemia, chemosis, follicular reaction, subconjunctival hemorrhage and membrane or pseudomembrane formation 6. Laboratory confirmation of the diagnosis can aid physicians in rapidly initiating suitable hygienic measures and determining the epidemiological significance of the infection.

A complication of viral conjunctivitis is the presence of multifocal subepithelial corneal infiltrates 7, which are focal lesions that may represent a cellular immune reaction against viral antigens that are deposited in the corneal stroma under the Bowman's membrane 8. These subepithelial infiltrates can persist for weeks to years and they may cause visual impairment if the infiltrate area involves the visual axis. Most of these infiltrates tend to resolve spontaneously without scarring. The use of topical corticosteroids may hasten recovery, but it does not affect long-term outcomes 9.

Various methods can be used to diagnose viral infections in the laboratory, including viral culture, antigen detection, serology and nucleic acid detection. Nucleic acid detection is more sensitive than other techniques and is not dependent on the presence of a viable virus or the quality or presence of appropriately infected cells. Therefore, polymerase chain reaction (PCR) is now emerging as the “gold standard” for diagnosing viral conjunctivitis. Studies suggest that PCR is more sensitive for detecting adenoviruses than other virological methods.

In Brazil, epidemiological data that can be used to determine the prevalence of ocular infections that involve adenoviruses are scarce. The purpose of this study was to investigate the frequency of adenoviral conjunctivitis and the incidence of subepithelial corneal infiltrates in a group of patients treated at the Ophthalmology Emergency Room of the Hospital das Clinicas, University of Campinas, Brazil.

## MATERIALS AND METHODS

A prospective, nonrandomized clinical study was conducted to evaluate 122 consecutively enrolled patients who were treated at the Ophthalmology Emergency Room of the State University of Campinas from November 2011 through June 2012. Patients were enrolled within 1 week of developing signs and symptoms consistent with acute infectious conjunctivitis.

Ocular swab samples were collected from patients with conjunctivitis suspected to be caused by a human adenovirus (HAdV). The samples were placed in a sterile solution of 0.9% NaCl and maintained in a freezer at -80°C degrees until the time of extraction. These samples were used for adenovirus PCR analyses. Adenovirus primers were designed based on the hexon region of the DNA sequences of adenovirus types 2 and 5: Hadv1, 5′-GCCGCAGTGGTCTTACATGCACATC-3′ and Hadv2, 5&prime-CAGCACGCCGCGGATGTCAAGT-3&prime (product size = 300 bp) 10-13. These primers can be used to amplify multiple serotypes. Sequencing of this 300-bp fragment of the hexon gene permitted the identification of most of the adenovirus serotypes that are associated with acute conjunctivitis. In addition, patients were investigated for the presence of sub-epithelial corneal infiltrates on the tenth and thirtieth days after the initial evaluation.

Written informed consent was obtained from the study participants. This study was approved by the ethics committee of the State University of Campinas.

### Inclusion and Exclusion Criteria

Eligible patients were required to have had acute unilateral or bilateral viral conjunctivitis (with characteristic clinical features, such as the sudden onset of acute follicular conjunctivitis with watery discharge, hyperemia and chemosis) for less than one week. In addition, they were required to have at least one of the following features that are consistent with viral conjunctivitis: ipsilateral preauricular lymphadenopathy preceded by flu-like symptoms (including fever, malaise, respiratory symptoms, nausea, vomiting, diarrhea, or myalgia), and/or a recent history of an eye examination or exposure within the family or at work. Exclusion criteria included a history of seasonal allergic conjunctivitis, the use of ocular medication following the onset of symptoms, contact lens use, a history of herpetic eye disease, a history of ocular surgery, a history of chronic ocular disease other than refractive error, allergy to iodine, pregnancy, patients less than 18 years of age, any bleeding disorder, glaucoma, significant blepharitis or dry eyes according to a slit lamp examination, purulent ocular discharge, corneal epithelial fluorescein staining, or intraocular inflammation.

## RESULTS

The baseline characteristics of the 122 included patients are shown in Table 1 (showing all patients and patients with positive PCR results). Among the 122 consecutive patients with acute conjunctivitis, 72 (59.0%) were PCR positive for an adenovirus. The PCR results were negative for adenoviruses in 50 patients. No bacterial cultures were performed.

Seventeen patients (13.93%) developed subepithelial corneal infiltrates and all of these patients had a positive PCR result. These patients were successfully treated with topical prednisolone (0.1%) within four weeks.

## DISCUSSION

Conjunctivitis is the most frequent ocular disorder that is observed in ophthalmic clinics. Several viruses are associated with conjunctivitis, including members of the *Enterovirus* genus, particularly enterovirus 70 (EV70) and a variant of Coxsackievirus A24 (vCA24). However, adenoviruses are the leading cause of acute conjunctivitis 14,15.

Results from studies conducted in Japan showed that adenoviruses were involved in 90% of all viral cases of conjunctivitis in that country 16, whereas worldwide, adenoviruses have been found to be involved in 15% to 70% of all cases of infectious conjunctivitis 17-20. It is often difficult to clinically distinguish a disease caused by an adenovirus from other etiologies of conjunctivitis and comparison of laboratory studies of acute conjunctivitis shows that the accuracy of clinical diagnosis ranges from 40% to 75% 21,22.

Very few studies have been conducted in Brazil to investigate the prevalence of adenoviruses in patients with symptoms of acute conjunctivitis. Maranhao et al. studied 75 eye swabs and reported that 60% of the patients had positive PCR results for an adenovirus 23. We found that the prevalence of adenoviral conjunctivitis was 59% (72 out of 122) in all patients presenting with a clinical diagnosis of infectious conjunctivitis at the Ophthalmology Emergency Room of UNICAMP between November 2011 and June 2012. This result is in agreement with the findings of similar previous studies conducted in Brazil 23 and the United States 24. Possible reasons for negative PCR results include the presence of viral conjunctivitis caused by non-adenoviral species, allergic conjunctivitis, chlamydia and inclusion conjunctivitis. Less common causes include herpetic viruses, picornaviruses, Epstein-Barr virus, influenza viruses, paramyxovirus and poxviruses.

Our study describes an incidence of subepithelial infiltrates of 13.93%. However, previous studies have described an incidence of up to 50% 7. Because subepithelial infiltrates can cause visual impairment if they involve the visual axis and because they occur only in adenoviral conjunctivitis, laboratory confirmation of the presence of an adenovirus may indicate the need for closer monitoring and early treatment, if necessary, to reduce the risk of permanent visual deterioration.

A laboratory confirmation of an adenovirus-related etiology may aid the physician in making an accurate diagnosis. The correct identification of patients with adenoviral conjunctivitis may reduce the spread of the disease and limit its toxicity in addition to allergic reactions and antibiotic resistance associated with unnecessary empirical treatments. Udeh et al. found that the systematic use of such a test could reduce the costs related to the inadequate use of antibiotics in patients with EKC by $71.30 US dollars per patient 25. Overlooking a diagnosis of viral conjunctivitis also poses a serious problem because of the high risk of transmission of the contagion. In addition, adenoviral conjunctivitis is associated with significant complications, including subepithelial infiltrates 26, lacrimal drainage abnormalities 27 and symblepharon formation 28.

The present study demonstrates that a tertiary hospital in Brazil experiences a prevalence of adenoviral conjunctivitis that is similar to that observed in other parts of the world, but no laboratory tests are routinely performed at the clinical level in this hospital. With the advent of new laboratory techniques, such as PCR, the ability to make diagnoses by analyzing tear samples from the inferior palpebral fornix has improved dramatically. A rapid, inexpensive and accurate method for diagnosing adenoviral ocular infections is needed not only to limit the transmission of the virus within the community but also to avoid the expensive, unnecessary, and ineffective use of antibiotic therapies.

## Figures and Tables

**Table 1:** The Prevalence of Adenoviral Conjunctivitis at the Clinical Hospital of the State University of Campinas, Brazil.

**Demographic and Clinical Characteristics**		
	**All patients (n=122)**	**Adenovirus PCR + (n=72)**
Median age (years)	36.09 (±13.30)	34.86 (±12.83)
Male (%)	41.8	44.4
Median days with symptoms	2.05 (±1.30)	2.12 (±1.26)
Associated upper respiratory infection (%)	28.7	30.6
Follicles on inferior tarsal conjunctiva (%)	94.3	95.8
Preauricular node (%)	18.0	15.3
Contact with person with a red eye (%)	53.3	58.3
